# Application of near-infrared hyperspectral imaging to discriminate different geographical origins of Chinese wolfberries

**DOI:** 10.1371/journal.pone.0180534

**Published:** 2017-07-13

**Authors:** Wenxin Yin, Chu Zhang, Hongyan Zhu, Yanru Zhao, Yong He

**Affiliations:** College of Biosystems Engineering and Food Science, Zhejiang University, Hangzhou, China; Agricultural University of Athens, GREECE

## Abstract

Near-infrared (874–1734 nm) hyperspectral imaging (NIR-HSI) technique combined with chemometric methods was used to trace origins of 1200 Chinese wolfberry samples, which from Ningxia, Inner Mongolia, Sinkiang and Qinghai in China. Two approaches, named pixel-wise and object-wise, were investigated to discriminative the origin of these Chinese wolfberries. The pixel-wise classification assigned a class to each pixel from individual Chinese wolfberries, and with this approach, the differences in the Chinese wolfberries from four origins were reflected intuitively. Object-wise classification was performed using mean spectra. The average spectral information of all pixels of each sample in the hyperspectral image was extracted as the representative spectrum of a sample, and then discriminant analysis models of the origins of Chinese wolfberries were established based on these average spectra. Specifically, the spectral curves of all samples were collected, and after removal of obvious noise, the spectra of 972–1609 nm were viewed as the spectra of wolfberry. Then, the spectral curves were pretreated with moving average smoothing (MA), and discriminant analysis models including support vector machine (SVM), neural network with radial basis function (NN-RBF) and extreme learning machine (ELM) were established based on the full-band spectra, the extracted characteristic wavelengths from loadings of principal component analysis (PCA) and 2nd derivative spectra, respectively. Among these models, the recognition accuracies of the calibration set and prediction set of the ELM model based on extracted characteristic wavelengths from loadings of PCA were higher than 90%. The model not only ensured a high recognition rate but also simplified the model and was conducive to future rapid on-line testing. The results revealed that NIR-HSI combined with PCA loadings-ELM could rapidly trace the origins of Chinese wolfberries.

## Introduction

Chinese wolfberry is a multi-branched shrub in the family Solanaceae, and the fruit, skin, and leaves can be used as medicine [1]. What’s more, the wolfberry shrubs are widely planted in Inner Mongolia, Shaanxi, Gansu, Ningxia, Qinghai and Sinkiang and other places in China for it has excellent soil and water conservation capacity [2]. It is well accepted that the growing environment may alter the chemical composition and biological properties of a selected botanical [3]. Most consumers favor the Ningxia wolfberries, which have a characteristic large fruit, nice shape, high content of active ingredient and a wide range of medicinal value [1]. However, with the frequent mixing of fruits from different origins in the market in recent years, the quality of Ningxia wolfberries is difficult to guarantee. According to most researches, the geographical origins of Chinese wolfberries can be identified based on observing the shape of the wolfberries and using chemical methods to detect internal quality, however, these methods are time-consuming, destructive to the samples and with low detection accuracy [4–5]. Therefore, establishing rapid, nondestructive and high-accurate methods to trace the origin of Chinese wolfberries is urgent. Meanwhile, these analytical methods are also needed for wolfberry breeding efforts to obtain improved cultivars with enhanced nutritional and nutraceutical quality and farm gate value for commercial production of Ningxia wolfberries [6].

Spectroscopic and spectral imaging techniques have been widely used to identify origins and analyze quality of agricultural products as rapid, nondestructive testing methods in recent years [7–8]. Near infrared reflectance spectroscopy (NIRS) which is based on the absorption of electromagnetic radiation in the 780–2526 nm wavelength range can provide comprehensive structural information on the components and properties of samples at the molecular level. It’s proven that this region of spectral bands arises from overtones of C-H, C-O, O-H and N-H stretching vibrations [9]. Several studies have been reported of using NIRS with chemometrics methods to determinate the wolfberry origins and quality. Wang [10] et al. (2016) used near-infrared diffuse reflectance spectroscopy (NIDRS) to evaluate the amount of Chinese wolfberry polysaccharides (LBPs). Li [11] et al. (2017) used a fourier transform near-infrared (FT-NIR) spectrometer to determine the total sugar content of Chinese wolfberry. Shen [12] et al. (2016) used NIRS to determinate geographical origins of Chinese wolfberries and flavonoids content related to origins. Li [13] et al. (2017) used NIRS to determinate geographical origins and anthocyanin content of black goji berry. They all concluded that NIRS has a high potential for determination of wolfberry origins and quality. However, the problem is associated with these methods, that samples were damaged when crushed into powder, making visual recognition difficult. Furthermore, although NIRS could obtain the internal quality information of samples from the spectra, NIRS fails to provide external space information of the samples.

Spectral imaging is the integration of spectroscopy and digital imaging, obtaining the spectral and spatial information of the object simultaneously. The near infrared hyperspectral imaging (NIR-HSI) is one of the common forms of the spectral imaging. It can obtain a wider range of internal and external information of the sample, leading to a more comprehensive analysis [14], which is helpful in discriminating different geographical origins of Chinese wolfberries. By hyperspectral imaging system, one pixel of each hyperspectral image has a wavelength covering the whole spectral range. Finally, a hyperspectral cube, which is composed of a series of images at each wavelength, is generated. NIR-HSI has been successfully used to discriminate origins and quality of some agricultural products. Marena [7] et al. (2011) used NIR-HIS to examine single whole kernels of three cereals (barley, wheat and sorghum) with varying topographic complexity. Paul [15] et al. (2016) used NIR hyperspectral imaging to classify maize kernels of three hardness categories with two approaches, pixel-wise and object-wise, however, in their research, three categories and 20–40 kernels of each category were insufficient to establish robust discriminative models and characteristic wavelengths were missing to simplify the models. Stephen [16] et al. (2013) used the near-infrared hyperspectral technique to measure the flour yield, softness and sucrose content of wheat and achieved a reliable evaluation of wheat milling quality. Gao [17] et al. (2013) used a pushbroom hyperspectral imaging system to discriminate different geographical origins of Jatropha curcas L. seeds by spectral and image processing technique respectively. Few papers did research on qualitative and quantitative analysis of Chinese wolfberry origins using NIR-HSI.

In this research, four geographical origins of Chinese wolfberries were studied using NIR-HSI technique. After acquisition of the hyperspectral data for Chinese wolfberries, all the spectral information of all samples was extracted. First, principal component visualization analysis was conducted on Chinese wolfberries from different areas in the pixel-wise approach. Then, the mean spectra of the wolfberry samples were analyzed to build support vector machine (SVM), neural network with radial basis function (NN-RBF) and extreme learning machine (ELM) models. Additionally, the characteristic wavelengths were selected to rapidly identify wolfberry origin from loadings of principal component analysis (PCA) and 2nd derivative spectra. The primary purpose of this research was to study the feasibility of using NIR-HSI to trace the origins of Chinese wolfberries and build the corresponding robust discriminating models using chemometric methods.

## Materials and methods

### Samples and sample preparation

**Fig 1:**
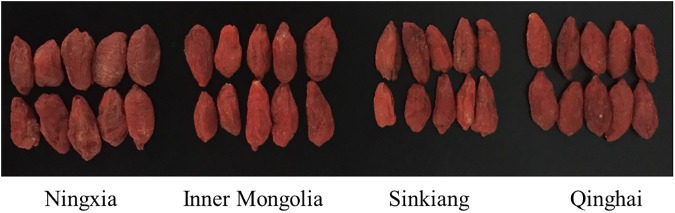
RGB image of the Chinese wolfberries from four different geographical origins. From left to right, followed by Ningxia, Inner Mongolia, Sinkiang and Qinghai.

To ensure that geographical origin was the only experimental variable, the wolfberry samples were collected from the same species called *lycium barbarum*. In this study, the wolfberries were purchased from local farmers in four main producing areas, including Zhongning County (105.67°E, 37.48°N, Zhongwei, Ningxia, China), Urad Front Banner (108.65°E, 40.72°N, Bayan Nur, Inner Mongolia, China), Jinghe County (82.88°E, 44.60°N, Bortala Mongolia, Sinkiang, China), Dulan County (98.08°E, 36.30°N, Haixi, Qinghai, China). The wolfberry shrubs are widely planted in Ningxia, Inner Mongolia, Sinkiang and Qinghai in China by local farmers. We could use these wolfberries as food as well as do studies on them. So, no specific permissions were required for these locations. In addition, we have confirmed that the field studies did not involve endangered or protected species. A single wolfberry was used as a sample. From each producing area, 300 samples were selected, and a total of 1200 samples were collected. To determine the origin of wolfberries by approximation of value assignment, Ningxia was assigned to 1, Inner Mongolia was assigned to 2, Sinkiang was assigned to 3 and Qinghai was assigned to 4. The surface of each wolfberry sample was wiped clean, and the samples were tiled separated from one another on the hyperspectral instrument platform. The captured RGB images of the Chinese wolfberries from the four different origins are shown in Fig 1, with no obvious difference in appearance.

### Hyperspectral image acquisition

**Fig 2:**
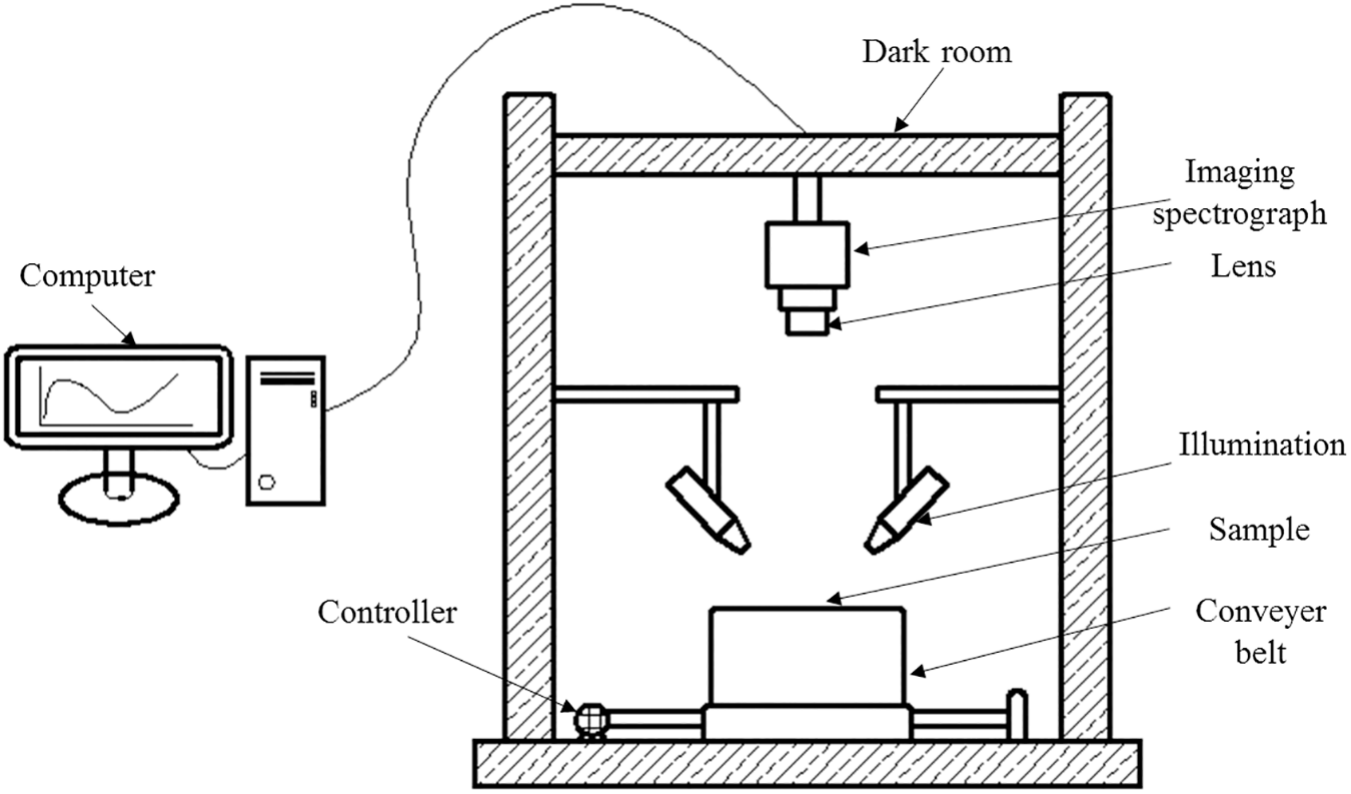
Schematic diagram of the primary components of the hyperspectral imaging device. All primary components were listed.

A laboratory-based hyperspectral imaging system (Fig 2) was used to measure and collect the information on wolfberries. The system consisted of a N17E-QE imaging spectrometer (Spectral Imaging Ltd., Oulu, Finland), C-mount imaging lens (OLES22; Specim, Spectral Imaging Ltd., Oulu, Finland), two 150 W tungsten halogen lamps (Fiber-Lite DC950 Illuminator; Dolan Jenner Industries Inc., Boxborough, MA, USA) placed on each side of the camera symmetrically at a 45° angle for illumination, an IRCP0076 Electronically Controlled Displacement Platform (Isuzu Optics Corp., Taiwan, China), a computer and a black box. Five nanometers was the spectral resolution. When collecting hyperspectral images, the black box was closed to avoid the interference from external light.

During hyperspectral image acquisition, the exposure time of the camera, the speed of platform movement and the distance between the lens and the sample were set. These three parameters affected one another, and the purpose of parameter adjustments was to produce sizeable, clear, ametabolic and undistorted collected images. After repeated attempts, the distance between the lens and the sample was set to 31 cm, the exposure time was set to 4 ms, and the speed of platform movement was set to 18.3 mm/s. The resolution of the near-infrared hyperspectral image was 320 × 256 pixels. Image acquisition software was provided by Taiwan Wuling Optics Company. Images were processed with ENVI 4.6 (ITT Visual Information Solutions, Boulder, Utah, USA) and MATLAB 7.12 (The Math Works, Natick, MA, USA). Before image processing, the acquired spectral images required correction, and the image correction was conducted using the following equation, where *I*_*c*_ is the corrected image, *I*_*raw*_ is the raw image, *I*_*white*_ is the white reference image, and *I*_*dark*_ is the dark reference image. The spectrum of the hyperspectral image had a corresponding relationship with the image. In this research, the full-pixel spectra of all Chinese wolfberries were extracted, and the spectral mean of all pixels of each Chinese wolfberry was used as the average spectrum of a sample.

$$I_{c}=\frac{I_{raw}-I_{dark}}{I_{white}-I_{dark}}$$ (1)

### Data analysis

#### Characteristic wavelengths selection

Much redundant and collinear information occurs within the spectral information, which greatly disturbs the extraction of effective spectral information. Moreover, abundant spectral data cause models to be complex and the calculations therefore time consuming. In this research, loadings of principal component analysis (PCA loadings) and 2nd derivative spectra were used to select the characteristic wavelengths to reduce the influence of redundant and collinear information, simplify the model and reduce the computational burden.

The loadings of principal component analysis reflect the degree of correlation between the principal components and the raw wavelength variable. Larger loadings of the principal component analysis indicate greater importance of the corresponding wavelength variable, with more information contained [18–19]. To select the characteristic wavelengths by PCA loadings, the contribution rate of different principal components (PCs) was determined, and then the cumulative contribution rate of analyzed PCs and the number of PCs were selected. Then, to determine the loadings of the corresponding PCs, the threshold was set and the peaks or valleys were selected based on the wavelength-loading map as the characteristic wavelengths.

One of the commonly used methods of spectral preprocessing is derivative spectra, which can effectively highlight the characteristic information of the spectrum [20]. Derivative spectra are used to select the characteristic wavelength [21–22] by selecting the appropriate peaks or valleys. In this research, the characteristic wavelength was selected based on the second derivative spectra. Because noise greatly influenced the derivative spectrum, before selecting the characteristic wavelengths, the raw spectra were smoothed by Savitzky-Golay [23] (SG) smoothing to minimize the noise of the raw spectrum.

#### Discriminant analysis methods

In this research, first, the principal component analysis (PCA) visualization analysis of all-pixel spectral information of Chinese wolfberry from four different areas was conducted. Then, the support vector machine (SVM), neural network with radial basis function (NN-RBF) and extreme learning machine (ELM) discriminant analysis models were established based on the average spectral information of all samples.

PCA [24] is an effective algorithm to solve the problem of data multicollinearity, extract feature information of data and realize data compression. PCA transforms multiple variables into a new coordinate system by linear transformation, and the largest variance of the data is projected on the first coordinate (the first principal component, PC1), the second largest variance is projected on the second coordinate (the second principal component, PC2), etc., to obtain the same number of principal components as the number of variables. In this research, the first five principal components were selected according to the cumulative contribution rate, and the scores were graphed. By combining the score information and the spatial information of spectral variables, the principal component was visualized.

SVM [25–27] is a statistical learning method based on structured risk minimization. With SVM, the sample space is mapped to a high-dimensional or infinite-dimensional feature space by nonlinear mapping. Linear partitioning or regression is achieved in a high-dimensional feature space by a linear hyperplane. This method can solve the problems of fewer samples, nonlinearity and high dimensions and overcome the local minimum in the neural network. In this study, different penalty parameters (c) and kernel function parameters (g) were chosen to achieve the highest recognition rate.

NN-RBF [28] is a 3-layer feed-forward neural network, which has the advantages of fast training speed, great generalization ability and arbitrary approximation. The purpose of the learning of NN-RBF is to determine the number of hidden layer neurons, the category of NN-RBF function, center and width and then determine the weight between the hidden layer and the output layer. In this research, by setting the rate of spread in the NN-RBF neural network to 0.1–1 and 1–100, the model achieved the highest recognition rate, and the value of spread when the recognition rate of the model was the highest was selected as the best parameter.

ELM [29] is an artificial neural network model proposed by Huang et al. The optimal solution is obtained by setting the number of neurons in the hidden layer and by comparing the effects of the different numbers of neuron nodes. In this research, the number of neurons in the hidden layer was optimized from 1 to 150 in steps of 1, and the number of neurons under the minimum training error was the number of hidden layer neurons in the ELM model.

## Results and discussion

### Pixel-wise analysis and classification

**Fig 3:**
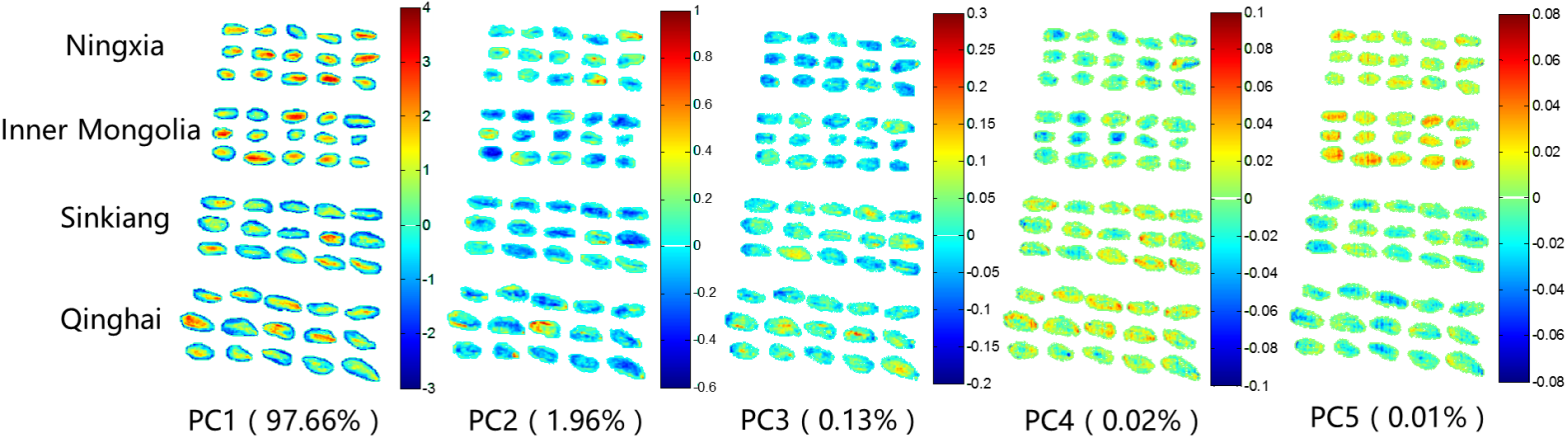
Score images for the first five principal components. The changes of color represent internal distributions in Chinese wolfberries from four different origins.

**Fig 4:**
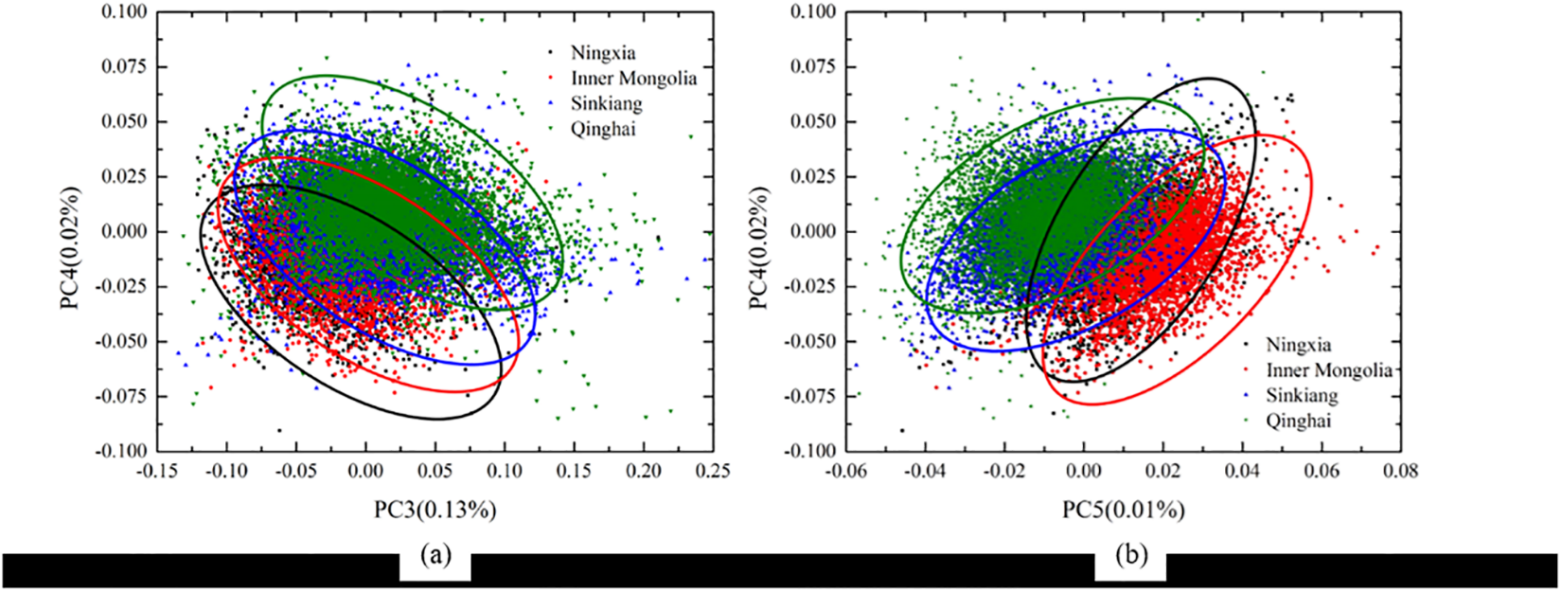
**Pixel-wise 2D PCA scores scatter plots of (a) PC3 and PC4 and (b) PC5 and PC4.** Samples from the same origin were more concentrated, forming four different regions.

To visualize the difference among Chinese wolfberries from the four geographical origins, PCA was conducted of all-pixel spectral information of Chinese wolfberries from the four different areas. In this research, the background and the insignificant pixels were eliminated, and the spectral information of 20,196 pixels of Chinese wolfberries from four locations was obtained, which was followed by PCA. The first five PCs were determined, and the scores of the PCs were plotted on the basis of the scores of each pixel and the spatial distribution of the pixels (Fig 3). In Fig 3, different colors represent different scores. The cumulative contribution rate of the first five principal components was 99.78%, which explained most of the spectral variables. As shown in Fig 3, in the score image for PC1, the types of color distribution are clearly different for the top two locations and the two below. In the score image for PC2, the top location was warmer in color than the remaining locations, which were cooler in color. Although the rates of contribution of PC3, PC4 and PC5 accounted for only a very small part of the total, they contained more internal information that could characterize different areas of origin. From their scoring charts, the internal distributions of Chinese wolfberries from different origins were different. In the score images for PC3 and PC4 (Fig 3), the color distribution gradually changed from a cool to warm tone for the wolfberries of different origins from top to bottom. For PC5, the color distribution gradually changed from a warm to cool tone for the wolfberries of different origins from top to bottom in the score image (Fig 3). Because the origins were easier to distinguish in the score images for PC3, PC4 and PC5, the score distribution was plotted with the scores of those three principal components (Fig 4). The scores of principal component analysis often show the intrinsic information of samples. Although the score distribution map of Chinese wolfberries from different origins overlapped with one another (Fig 4), samples from the same origin were more concentrated, forming different regions. Thus, although the differences among the Chinese wolfberries from four origins could be reflected intuitively, accurate classification was not easily achieved.

### Object-wise analysis and classification

#### Spectral features of Chinese wolfberries from different geographical origins

**Fig 5:**
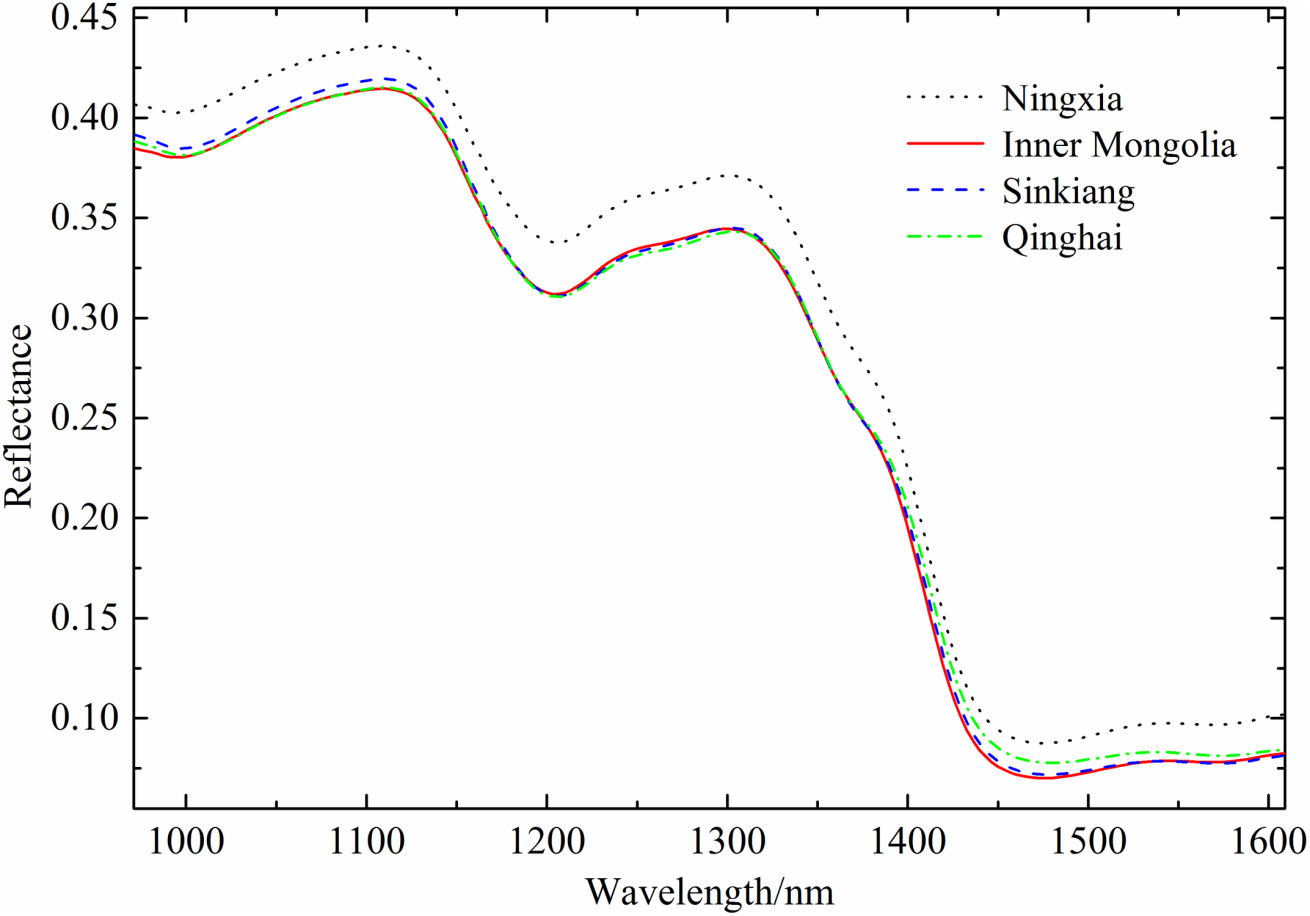
Mean reflectance spectra of wolfberries from different geographical origins in the range of 972–1609 nm. The noise from the front and back ends of the spectral curves was removed, and the spectra were pretreated with moving average smoothing (MA).

In the object-wise approach, the depicted objects (in this case Chinese wolfberries) were used as data points instead of the individual pixels. The spectral reflectance of all pixels of each Chinese wolfberry was averaged as the spectral reflectance of a sample, and a total of 1200 spectral curves were obtained. The noise from the front and back ends of the spectral curves was removed, the spectra pretreated with moving average smoothing (MA) in the range of 972–1609 nm were selected for analysis. The mean spectra of Chinese wolfberries from four different geographical origins are shown in Fig 5. The wolfberries from different geographical origins had similar spectral patterns and all had absorption peaks at approximately 995, 1200 and 1465 nm. The absorption peak near 995 nm was attributed to the second vibration of N-H bonds in proteins or amino acids [30–31]. The absorption peak near 1200 nm was attributed to the secondary stretching vibration of C-H bonds in starch, proteins or lipids [32–33]. The absorption peak near 1465 nm was the sensitive region for water absorption [34]. As shown in Fig 5, the average spectra in the range of 972–1609 nm for the Chinese wolfberries from four different geographical origins showed similar spectral curves and slightly different reflectance values. The feature might be caused by differences in the internal components based on different regions and climates.

#### Object-wise principal component analysis

**Fig 6:**
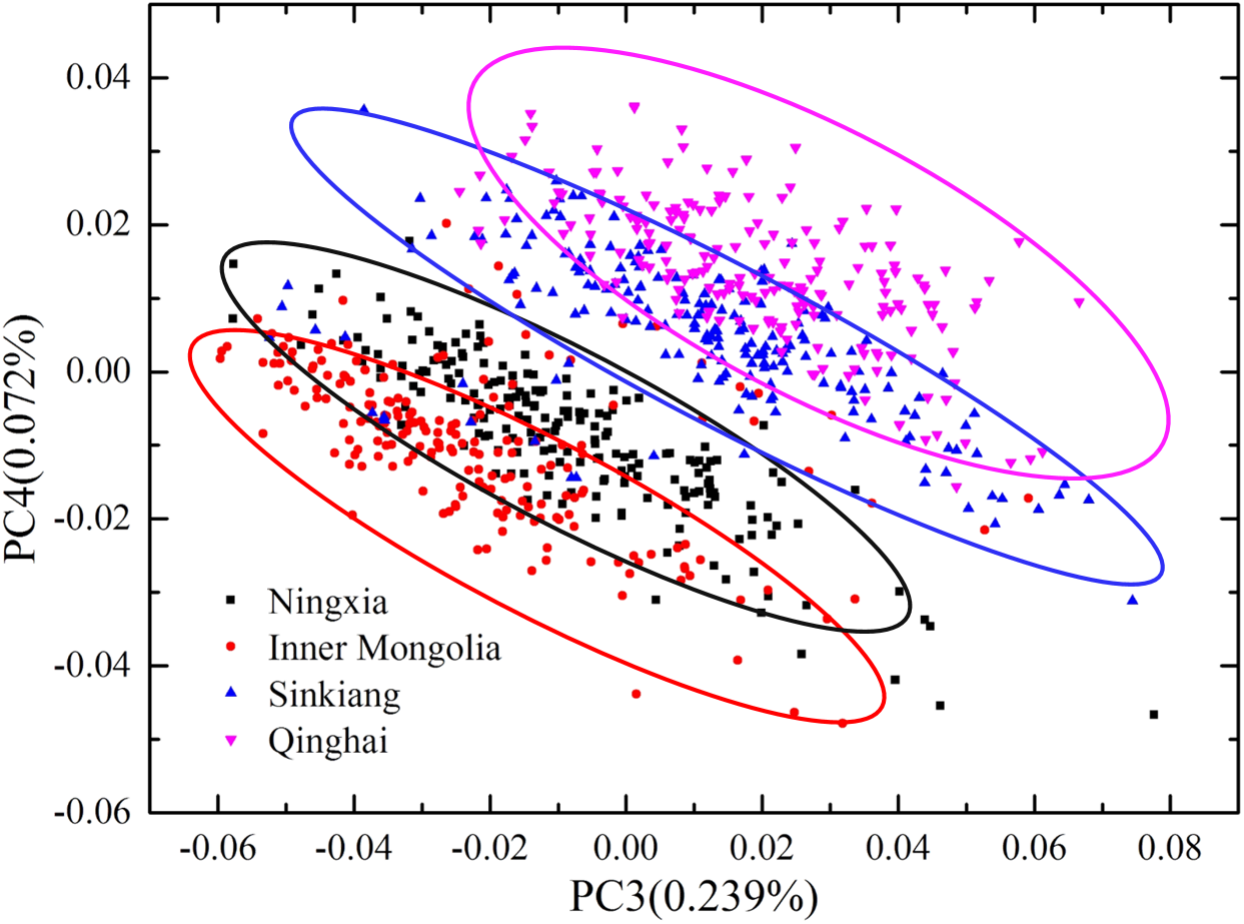
Object-wise 2D PCA scores scatter plot of PC3 and PC4. Samples from each geographical origin were clustered together by their own characteristics.

The spectral data of 1200 samples from four geographical origins were divided into a calibration set and a prediction set according to the Kennard-Stone algorithm [35] at a ratio of 2:1, with 200 samples from each geographical origin used as the calibration set and remaining 100 samples from each geographical origin used as the prediction set. PCA was performed on the spectral data of the modeling set for qualitative analysis of differentiating geographical origins of Chinese wolfberries. Fig 6 shows the 2D scores scatter plot for PC3 and PC4. As shown in Fig 6, the samples from each geographical origin were clustered together by their own characteristics, although some overlaps remained in the score map. Further analysis and processing were required to identify the different origins of Chinese wolfberries.

### Discriminant models based on full spectra

The discriminant analysis models of wolfberry origins were established by spectral data pretreated with MA. Support vector machine (SVM), neural network with radial basis function (NN-RBF) and extreme learning machine (ELM) were used to establish the discriminant models. Table 1 shows the recognition results of the models.

**Table 1:** Classification results of discriminant models based on full spectra.

Model	Parameter^a^		Accuracy/%									
			Calibration					Prediction				
			1	2	3	4	Mean	1	2	3	4	Mean
SVM	(c, g)	(256, 0.0118)	96.00	88.00	86.00	94.00	**91.00**	99.00	83.00	76.00	95.00	**88.25**
NN-RBF	s	25	100	92.50	96.00	99.50	**97.00**	98.00	87.00	79.00	97.00	**90.25**
ELM	h	162	100	92.50	93.50	99.00	**96.25**	97.00	86.00	83.00	99.00	**91.25**

1: Ningxia; 2: Inner Mongolia; 3: Sinkiang; 4: Qinghai

^a^c: the penalty parameter of the SVM model; g: the parameter of kernel function; s: the spread rate of the NN-RBF model; h: the hidden node number of the ELM model.

Among the models, the penalty parameter (c) of the SVM model was 256, the parameter of kernel function (g) was 0.0118, the spread rate (s) of the NN-RBF model was 25, and the hidden node number (h) of the ELM model was 162. The recognition accuracies of wolfberries from Inner Mongolia and Sinkiang were all less than the mean accuracies. ELM and NN-RBF models achieved better discriminant effects, with the recognition accuracies of the calibration set higher than 95% and those of the prediction set higher than 90%. The recognition effect of the SVM model was the worst, with 91% recognition accuracy of the calibration set and 88.25% for that of the prediction set. Although the number of samples was large, the recognition accuracies of the calibration set and prediction set of all identification models exceeded 85%. Thus, it’s feasible to discriminate geographical origins of Chinese wolfberries using full spectra.

#### Characteristic wavelengths selection

Although the models based on the full spectra had good results, the spectral data of 190 bands increased the computational complexity, in addition, the redundant information and collinearity of the spectral data might affect the effectiveness of the established models. Therefore, loadings of PCA and 2nd derivative spectra were used to select the characteristic wavelengths and extract the effective feature information in the spectra to build the identification models of geographical origins [17].

**Fig 7:**
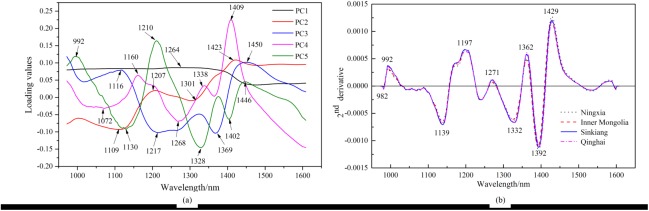
**Selected optimal wavelengths by loadings of principal component analysis (a) and 2nd derivative spectra (b).** All characteristic wavelengths were marked.

When the characteristic wavelengths were selected by loadings of PCA, the loadings of the first five PCs were selected according to the requirement of the cumulative contribution rate. The wavelength-loading plot was drawn by the loadings of the first five PCs, and the peaks or valleys were selected as the characteristic wavelengths. A total of 20 characteristic wavelengths were selected, including 992, 1072, 1109, 1116, 1130, 1160, 1207, 1210, 1217, 1264, 1268, 1301, 1328, 1338, 1369, 1402, 1409, 1423, 1446, and 1450 nm (Fig 7(A)). The peaks or valleys based on the wavelength-loading map were indicative of physical and chemical effects involving [22] fat, carbohydrate, protein and water in the wolfberries. Before the characteristic wavelengths were selected by 2nd derivative spectra, smoothing with SG minimized the noise of the raw spectra. Then, the second derivative spectra were plotted, and the peaks or valleys were selected as the characteristic wavelengths. From the second derivative spectra, a total of nine characteristic wavelengths were selected, including 982, 992, 1139, 1197, 1271, 1332, 1362, 1392, and 1429 nm (Fig 7(B)). The detailed biophysical attributes of these 29 wavelengths were listed in Table 2 [36–41].

**Table 2:** Attributes of selected characteristic wavelengths.

Characteristic wavelengths (nm)	Biophysical attributes
982, 1160, 1268, 1271, 1369, 1369, 1402, 1409, 1446	O-H stretch of water
992	O-H stretch of fat
1130, 1139, 1207, 1338, 1392	C-H stretch of fat
1197, 1300	C-H stretch of the carbohydrate
1210, 1217, 1328, 1332, 1362	C-H stretch of fat, carbohydrate, protein
1072, 1109, 1116, 1264, 1423, 1429, 1450	N-H stretch of protein

#### Discriminant models based on characteristic wavelengths

Models of SVM, NN-RBF and ELM were established based on the characteristic wavelengths extracted by PCA loadings. Table 3 shows the recognition effect of each model for the calibration set and the prediction set. The penalty parameter (c) of the SVM model was 256, the parameter of kernel function (g) was 16, the spread rate of the NN-RBF model (spread) was 3, and the number of nodes of the hidden layer of the ELM model was 89. The SVM, ELM and NN-RBF models all achieved good recognition results for the large sample size, and the recognition accuracies of the calibration set were above 90%, and the accuracies of the prediction set were above 85%. The SVM model achieved the worst recognition effect, and the accuracy of the calibration set was 90.75% and that of the prediction set was 86.5%. The recognition accuracies of the NN-RBF calibration set and prediction set were 94% and 90%, respectively, which were the best of the models based on the characteristic wavelengths extracted by PCA loadings.

**Table 3:** Classification results of discriminant models based on characteristic wavelengths.

Model	PCA loadings				2nd derivative spectra			
	Parameter^a^		Mean Accuracy/%		Parameter		Mean Accuracy/%	
			Calibration	Prediction			Calibration	Prediction
SVM	(c, g)	(256, 16)	90.75	86.50	(c, g)	(256, 1)	89.38	82.75
NN-RBF	s	3	94.00	90.00	s	6	94.38	85.50
ELM	h	89	93.25	90.00	h	172	93.75	86.00

^a^c: the penalty parameter of the SVM model; g: the parameter of kernel function; s: the spread rate of the NN-RBF model; h: the hidden node number of the ELM model.

Models of SVM, NN-RBF and ELM were also established based on the characteristic wavelengths extracted by the second derivative spectra. Table 3 shows the recognition effect of each model for the calibration set and the prediction set. The penalty parameter (c) of the SVM model was 256, the parameter of kernel function (g) was 1, the spread rate of the NN-RBF model (spread) was 6, and the number of nodes of the hidden layer of the ELM model was 172. Of the models, the NN-RBF and ELM models achieved better discriminant effects, and the recognition accuracies of the calibration set were higher than 90%, and the accuracies of the prediction set were higher than 85%. The SVM model achieved the worst recognition effect, with the recognition accuracy of the calibration set 89.375% and the recognition accuracy of the prediction set 82.75%. The ELM model obtained the best recognition effect, with the accuracy of the calibration set and the prediction set 93.75% and 86%, respectively.

#### Comparison of three models based on different datasets

The discriminant analysis models based on characteristic wavelengths extracted by PCA loadings and 2nd derivative spectra were compared with those based on full spectra. All the recognition accuracies of the calibration sets and the prediction sets of models exceeded 85%, except for the SVM model based on characteristic wavelengths extracted from 2nd derivative spectra. Although the selection of characteristic bands simplified the models, the discriminant models based on the characteristic bands were slightly inferior to the discriminant model based on the full spectra, and the discriminant analysis model based on the characteristic wavelengths extracted by PCA loadings was better than the discriminant analysis model based on the characteristic wavelengths extracted by the second derivative spectra overall. For the predictive effects of the models, the accuracies of the ELM prediction sets were higher than those of the other two models, and the models established by ELM algorithm achieved the best discriminant effects under the three processing conditions. In this research, the number of samples was 1200, which was large enough, and the recognition accuracies of the calibration sets and the prediction sets all exceeded 80%, which demonstrated the feasibility of discriminating different geographical origins of Chinese wolfberries using the HSI technique combined with a discriminant analysis model.

## Conclusions

The origins of Chinese wolfberries were traced using an NIR-HSI system combined with extracted characteristic bands and different discriminant analysis models. From the perspective of the pixel spectra of the Chinese wolfberries combined with the spatial distribution of the Chinese wolfberries, a principal component pseudo-color map was drawn, and the differences of wolfberries from four origins were displayed intuitively. From the perspective of wolfberry samples, different discriminant analysis models were built on the full spectra and the characteristic wavelengths extracted by PCA loadings and 2nd derivative spectra. Following analysis and comparison, the discriminant models based on the full spectra were better than those based on the characteristic wavelengths. Among the discriminant analysis modeling methods, ELM algorithm obtained the best discriminant effects. ELM model based on the characteristic wavelengths extracted by PCA loadings not only provided high recognition accuracy but also simplified the model, which facilitated rapid on-line detection. In future research, as many origins as possible of Chinese wolfberries should be studied to establish a more robust and wider range of identifications in models of the origins of Chinese wolfberries, and the feasibility should be studied of applying HSI technique to detect quality in Chinese wolfberry and determine whether Chinese wolfberry has been artificially smoked.

## Supporting information

S1 Dataset Calibration_ data. The original data of calibration set for object-wise analysis.
(XLSX)

S2 Dataset Pixelwise_ data. The hyperspectral data for pixel-wise analysis.
(XLSX)

S1 Fig Spectral reflectance curves. Mean reflectance spectra of wolfberries from different geographical origins in the range of 972–1609 nm with standard error bars.
(TIFF)
