# Supplementary figures and images for: Application of near-infrared hyperspectral imaging to discriminate different geographical origins of Chinese wolfberries
Source: PLoS One. 2017 Jul 13;12(7):e0180534. doi: 10.1371/journal.pone.0180534 (PMC5509235; doi:10.1371/journal.pone.0180534)

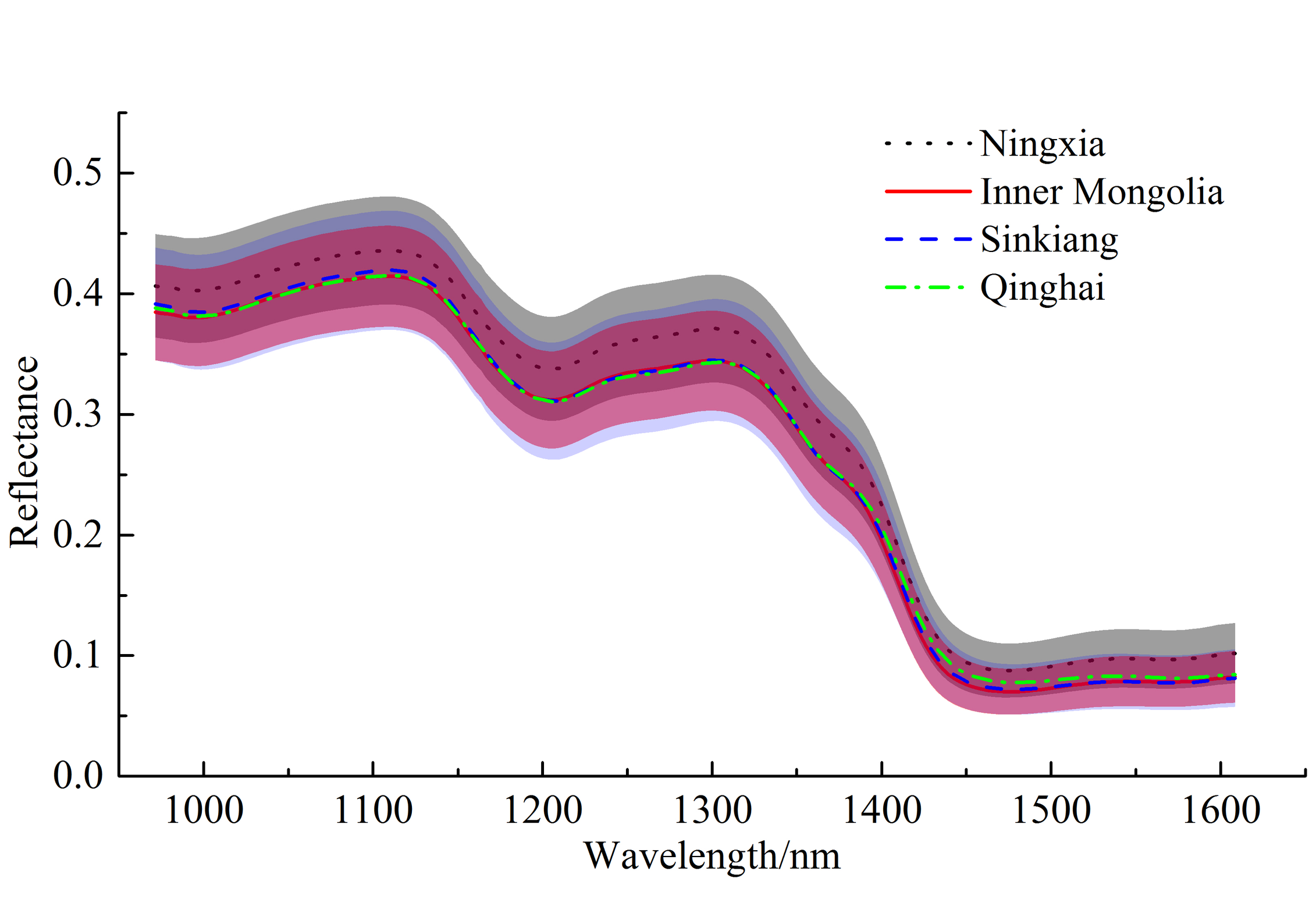

Supplement: S1 Fig — Mean reflectance spectra of wolfberries from different geographical origins in the range of 972–1609 nm with standard error bars. (TIFF) [file pone.0180534.s003.tiff]
